# Extrusion-Free Survival Following Glaucoma Drainage Device Surgery Using EverPatch Plus^®^: A Propensity Score-Weighted Survival Analysis

**DOI:** 10.3390/jcm15072570

**Published:** 2026-03-27

**Authors:** Etsuo Chihara, Tomoyuki Chihara, Leon W. Herndon

**Affiliations:** 1 Sensho-kai Eye Institute, Minamiyama 50-1, Iseda, Uji 611-0043, Kyoto, Japan; mail4chiharat@gmail.com; 2 Duke Eye Center, 2351 Erwin Rd, Box 3802, Durham, NC 27710, USA; leon.herndon@duke.edu

**Keywords:** anterior segment optical coherence tomography, artificial sclera, extrusion, EverPatch, glaucoma drainage implant, patch graft, polycarbonate urethane

## Abstract

**Objectives**: To evaluate extrusion-free survival following glaucoma drainage device (GDD) surgery using EverPatch Plus^®^ (EPP) and to compare outcomes with conventional scleral patch grafts using propensity score-based survival analysis. **Methods**: This retrospective case series included 19 eyes that underwent GDD implantation with EPP and 105 control eyes that received conventional scleral patch grafts. To adjust for baseline differences between groups, a propensity score for EPP use was estimated using multivariable logistic regression incorporating age, neovascular glaucoma, prior glaucoma surgery, preoperative intraocular pressure, number of glaucoma medications, quadrant of patch placement, and insertion site. Stabilized inverse probability of treatment weighting was applied. Because follow-up in the EPP group did not exceed 12 months, all survival analyses were performed with administrative censoring at 12 months. Extrusion-free survival was evaluated using Kaplan–Meier analysis and Cox proportional hazards modeling. **Results**: Within 12 months, patch extrusion occurred in 3 of 19 eyes in the EPP group and in 12 of 105 eyes in the scleral patch graft group. After inverse probability weighting, estimated 12-month extrusion-free survival was 83.5% in the EPP group and 88.4% in the scleral patch graft group, indicating no statistically significant difference between groups (log-rank test, *p* = 0.498). In an inverse probability-weighted Cox model, EPP use was not significantly associated with extrusion risk (hazard ratio ≈ 1.3; 95% confidence interval ≈ 0.4–4.0). **Conclusions**: After adjustment for baseline covariates and restriction of follow-up to 12 months, extrusion-free survival following glaucoma drainage device surgery using EPP was comparable to that achieved with conventional scleral patch grafts.

## 1. Introduction

To prevent extrusion of the silicone tube of glaucoma drainage device (GDD), various biological materials such as donor sclera, autologous sclera, pericardium, fascia lata, cornea, dura mater, KeraSys (IOP Inc., Costa Mesa, CA, USA), and amniotic membrane were employed as patch grafts to cover the tube [1,2,3,4,5]. Among these, donor sclera and pericardium were commonly used because of their favorable conformity to surrounding tissues and cost-effectiveness.

While biological patch grafts offer advantages such as suitable rigidity, elasticity, toughness, affordability, and ease of handling, their typical thickness of 400 to 500 μm may create a cosmetically noticeable elevation of the overlying conjunctiva. This protrusion may induce progressive thinning or atrophy of the overlying tissues, eventually leading to patch graft exposure. Furthermore, biological grafts are biodegradable and tend to thin over time [1,2,3,4], thereby increasing the risk of GDD exposure. They also carry potential risks of viral transmission and prion-related diseases, such as Creutzfeldt-Jakob disease [6,7].

To overcome the limitations associated with biological patch materials, the use of non-degradable synthetic material such as Polytetrafluoroethylene (PTFE; Gore-tex^®^, Teflon) [8,9], and high density polyethylene (Su-Por) have been investigated [10]. However, these material may induce a mild fibrotic reaction, [11] abscess formation [12], and have been associated with a relatively high prevalence of extrusion [10]. Although the biocompatibility of Styrene-block-isobutylene-block-styrene (SIBS) has been evaluated, this material has not been adopted for use as GDD patch grafts [13].

Polycarbonate urethane (PCU), a biocompatible and hydrophobic polymer, has shown promise in a range of biomedical applications. It has been used in artificial blood vessels [14], joint arthroplasty [15], heart valves [16], nerve wraps for peripheral nerve regeneration, gingival tissue engineering, drug delivery systems, cartilage reconstruction and more.

The material of CorNeat EverPatch Plus^®^ (EPP; CorNeat Vision, Ra’anana Israel) is a non-woven aromatic PCU sheet specifically designed for use in GDD surgeries to cover the drainage tube and prevent external protrusion. Its bio-affinity characteristics such as cytotoxicity, ocular irritation, systemic toxicity, and teratogenicity were evaluated according to ISO10993-6 (Biological evaluation of medical devices, ISO, Geneve, Switzerland, 1994) standards. These assessments were registered under the United States Clinical Trials Registry number NCT05469867 and conducted between 11 December 2020, and 1 July 2023. Histopathological findings have not been published in the peer reviewed literature; however, related information is available online [17].

**Figure 1:**
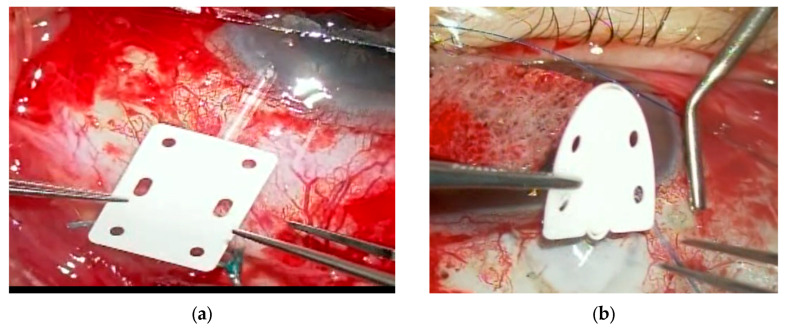
Intraoperative findings of rectangular-type (**a**) and Shield-type (**b**) EverPatch Plus^®^ (EPP) during Ahmed Glaucoma valve (AGV) implantation. The rectangular type (**a**) has a larger surface area and six fixation holes. In contrast, the Shield type (**b**) has a rounded anterior edge, smaller surface area, and four fixation holes.

Human testing, registered under NCT04037917, was conducted from 8 August 2022, to 18 June 2024, at DaVinci Eye Care in Tbilisi, Georgia. This study evaluated the clinical outcomes of 10 patients aged 18 to 80 years over a 12-month period, there was a 10% incidence of conjunctival penetration at the edges of the device. Finaly, this device received U.S. Food and Drug Administration (FDA) 510(k) clearance. A rectangular type measuring 0.5 cm × 0.65 cm and 100–150 μm thick, as shown in Figure 1a, and a Shield type featuring a smooth anterior edge (Figure 1b) were released in 2025.

Even when a new device receives approval from the FDA and is released to the market, unexpected complications may emerge in real-world clinical practice, potentially leading to criticism or product recall. In subsequent reports, extrusion of the EverPatch was observed in 13 of 29 eyes (approximately 45%) following implantation [18]. In such circumstances, it is essential to identify the underlying causes and consider appropriate countermeasures.

In the present study, we employed anterior segment optical coherence tomography (AS-OCT: CASIA II, Tomey Co., Aichi, Japan) to evaluate conjunctival thickness and associated clinical features. We used 16 Shield-type patches and three rectangular-type patches in our practice and we report their clinical performance over a 12-month follow-up period.

## 2. Subject and Methods

This interventional case series included nineteen eyes of nineteen patients who received EPP implantation. The prevalence of extrusion was compared with that in 105 eyes of 95 patients who received a human scleral patch at the Sensho-kai Eye Institute (Uji, Kyoto, Japan). Patients had severe glaucoma that was refractory to conventional glaucoma surgery such as trabeculectomy. All patients underwent GDD surgery by two surgeons (EC and TC): EC performed 97 surgeries in the control group and 14 in the EverPatch cohort, while TC, who exclusively used the Ahmed Glaucoma valve (AGV, New world Medical, Rancho Cucamonga, CA, USA) performed 8 surgeries in the control group and 5 in the EverPatch cohort. The EPP cohort underwent GDD surgery between January 2025 and May 2025. Eighteen patients were Japanese and one was Chinese. The control cohort includes a total of 105 Japanese eyes that underwent AGV FP7 implantation with a scleral patch between January 2020 and January 2025. The patch material was switched from scleral patch to EPP in January 2025.

Inclusion criteria were refractory glaucoma requiring GDD surgery, clear cornea, and absence of conjunctival erosion or active conjunctivitis prior to surgery. Patients with poor compliance and those who received a Baerveldt glaucoma implant (Johnson & Johnson, North Jacksonville, FL, USA) were excluded from the control cohort. The study adhered to the tenets of the Declaration of Helsinki and was approved by the institutional review board of Sensho-kai (C2025-01R, 2023-03R). Informed consent was obtained from all patients.

### 2.1. Anterior Segment Optical Coherence Tomography (AS-OCT) Evaluation

Patients were instructed to look downward or upward, and the light beam of the AS-OCT was directed perpendicular to the surface of the EPP. Conjunctival thickness was measured using the built-in measurement software of the AS-OCT system. Preoperatively, the deep episcleral vascular plexus often manifested as a hypo-reflective layer above the hyper-reflective sclera, allowing measurement of the conjunctiva-Tenon’s capsule complex. However, this hypo-reflective layer was indistinct in most postoperative cases. In such instances, the thickness was measured as the distance between the conjunctival surface and the surface of the EPP, which appeared as a white amorphous mass on AS-OCT.

This study focused on assessing the postoperative thickness of the conjunctiva-subconjunctival tissue complex over the EPP, the distance between the scleral spur and anterior edge of the EPP, as well as the presence of subconjunctival fluid accumulation, microcysts, and wound dehiscence. The effects of the EPP on conjunctival thickness were assessed at 1 mm and 3 mm from the anterior edge of the EPP; however, before surgery, where no patch material existed, measurements were taken at 1 mm and 3 mm from the scleral spur. These evaluations were conducted using AS-OCT, infrared slit-lamp imaging, and standard slit-lamp examination. When the distance between the scleral spur (SS) and the anterior edge of the EPP could not be measured due to poor-quality angle images, the corresponding eyes were excluded from the analysis.

AS-OCT CASIA II (mentioned above), Humphrey Visual Field Analyzer (Carl Zeiss Meditec Co., Tokyo, Japan), specular microscopy (CellCheck, Konan Co., Nishinomiya, Japan), Optos California (Nikon solutions Co., Tokyo, Japan), and Hamada’s infrared light Camera (Hamada-Shokai, Kyoto, Japan) were used to assess conjunctival findings, visual field defects, corneal endothelium, and infrared photographs of patch graft. Intraocular pressure (IOP) was measured using a Goldmann applanation tonometer (Haag-Streit Japan, Yokohama, Japan).

### 2.2. Statistical Analysis

Statistical analyses were performed using R software (version 4.5.2) and Bell Curve for Excel (Social Survey Research Information Co., Tokyo, Japan). Continuous variables were compared using analysis of variance (ANOVA), as appropriate.

### 2.3. Propensity Score and Weighting

A propensity score (PS) for receiving EPP was estimated using multivariable logistic regression. Baseline covariates were selected a priori based on clinical relevance and potential influence on patch selection (age, glaucoma subtype, previous ocular surgery, preoperative intraocular pressure, number of glaucoma medications, quadrant of tube insertion, and insertion site).

We applied overlap weighting to create a weighted pseudo-population that emphasizes patients with substantial clinical equipoise between treatment groups. Covariate balance was assessed using standardized mean differences (SMDs), with smaller absolute values considered indicative of adequate balance. The distribution of PS and weights was examined to confirm adequate overlap and to identify influential observations.

### 2.4. Time-to-Event Outcome

The primary endpoint was time to patch extrusion. Weighted Kaplan–Meier survival curves were generated, and between-group differences were evaluated using a weighted Cox proportional hazards model with robust (sandwich) variance estimation. Results are presented as hazard ratios (HRs) with 95% confidence intervals (CIs). As sensitivity analyses, stabilized inverse probability of treatment weighting (IPTW) was performed with truncation of extreme weights (e.g., at the 1st–99th percentiles).

The Wilcoxon signed-rank test was employed for paired comparison, and Haberman residual analysis was used for categorical data.

### 2.5. Surgical Techniques

Under local anesthesia, a radial incision and a fornix-based conjunctival incision was made at the limbus. In eyes with severe conjunctival scarring, a blunt knife was used with care to avoid conjunctival perforation. The tendons of two adjacent rectus muscles were exposed, and 4-0 silk traction sutures were placed. Subconjunctival scar tissue was excised to create adequate space for implantation of GDD. The plate of either an Ahmed glaucoma valve (AGV FP7; New world Medical, Rancho Cucamonga, CA, USA) or Paul Glaucoma Implant (PGI; Advanced Ophthalmic Innovations, Singapore) was positioned 8 mm posterior to the limbus and secured to the sclera using two 5-0 polyethylene sutures.

Before intraocular insertion, the tube of the AGV or PGI was passed through both the anterior and posterior loops of the EPP.

For tube insertion into the ciliary sulcus, viscoelastic material was injected beneath the iris to create space. A 23 G or 25 G needle was used to enter the sulcus through the sclera at a point 2.5 mm posterior to the limbus. For anterior chamber insertion, no viscoelastic was used; a disposable needle was used to enter 2.0 mm from the limbus, parallel to the iris plane. For pars plana insertion, the entry point was 3.5 mm posterior to the limbus.

The tube was then inserted into the ciliary sulcus, anterior chamber, or vitreous cavity through the pars plana, depending on the surgical plan.

The Shield type EPP had four fixation holes, which were threaded with 9-0 nylon sutures and firmly secured to the sclera. The suture knots were rotated and buried in the sclera. The rectangular-type EPP had six fixation holes; all were threaded and similarly secured to the scleral surface.

In the donor sclera cohort, a 3 × 5 mm scleral patch was placed over the tube. After inserting the tube into the anterior chamber, ciliary sulcus or vitreous cavity, the patch was firmly secured to sclera using four 9-0 nylon sutures.

The Tenon’s capsule and conjunctiva were carefully pulled forward and sutured at the limbus using a “locking” suture (combined running suture and single knot) with at least ten passes of 9-0 twisted polyglactin sutures. The surgical field was disinfected with povidone-iodine. Dexamethasone (0.4 mg) was injected, subconjunctivally, and the eye was dressed with ofloxacin and betamethasone ointment. Postoperative care included topical 0.3% gatifloxacin and 0.1% betamethasone, instilled four times daily for one month. Postoperative IOP was closely monitored, and anti-glaucoma medications were prescribed as needed.

During the preparation of this study, the authors used Chat GPT (version 5.2, Open AI) solely for English language editing and assistance with statistical programming. The AI tool was not used for data generation, data analysis, interpretation or scientific decision-making. The authors have reviewed and edited the output and take full responsibility for the content of this publication. 

## 3. Results

A total of 124 eyes were included in the analysis: 19 eyes received EPP and 105 eyes received scleral patch grafts. Baseline patient characteristics of the EPP cohort and the control scleral patch cohort are summarized in Table 1. In the EPP cohort, 19 eyes from 19 patients with refractory glaucoma underwent implantation of GDD: six with AGV and 13 with PGI. The Shield-type EPP was used in 16 eyes, while the rectangular type was used in three eyes. The tube was inserted into the ciliary sulcus in 11 eyes, the anterior chamber in six eyes, and the vitreous cavity in two eyes. The tube was placed in the superior temporal quadrant in 16 eyes and in the inferonasal quadrant in three cases. Following extrusion of the EPP, subsequent clinical data were excluded from statistical analysis. Seventeen cases were followed for more than 9 months, and the mean follow-up duration was 10.7 ± 1.0 months.

Intraoperative findings of the Shield type and rectangular type EPP are shown in Figure 1.

The clinical courses of the 19 eyes that underwent EPP implantation and the 105 eyes that received conventional scleral patch graft are summarized in Table 2. Differences in preoperative IOP between the EPP cohort and control group were not significant (*p* = 0.521 by ANOVA). Postoperative IOP at 3 months was lower in EPP cohort, in which PGI was used in 13 of the 19 eyes (Table 2).

### 3.1. Clinical Course of Conjunctival Thickness

**Figure 2:**
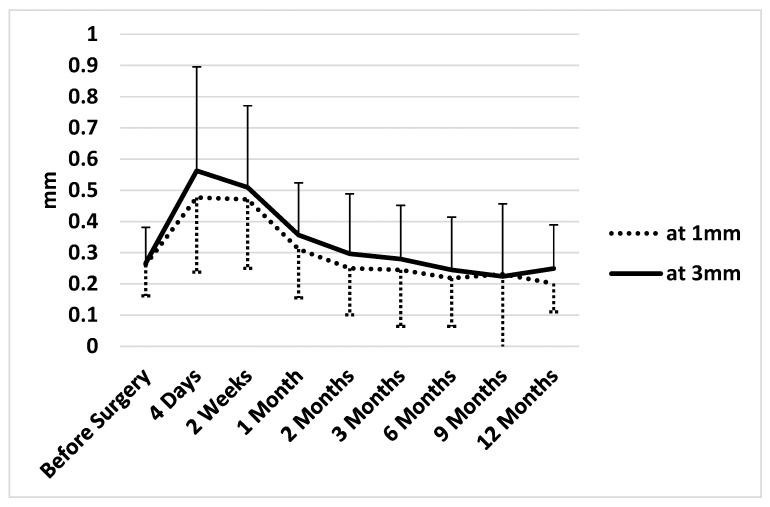
Changes in conjunctival thickness over time. Conjunctival thickness peaked on postoperative day 4 and decreased progressively thereafter. The increase was not statistically significant at 1 month or at subsequent time points.

Overall trends in conjunctival thickness, as assessed by anterior segment OCT (CASIA II), are summarized in Table 3 and illustrated in Figure 2. Eyes with a history of multiple prior surgeries demonstrated increased preoperative conjunctival thickness. Considerable inter-patient variation was observed in the early postoperative period, which gradually diminished over time.

Table 3 demonstrates a marked early postoperative increase in conjunctival thickness following EPP implantation. At 1 mm, thickness increased by 81.9% at 4 days (*p* = 0.002; Wilcoxon signed rank test) and remained elevated by 79.5% at 2 weeks (*p* = 0.001) compared with baseline. A similar pattern was observed at 3 mm, with increases of 110.8% at 4 days (*p* = 0.001) and 90.9% at 2 weeks (*p* = 0.001). Thereafter, conjunctival thickness progressively decreased toward baseline. By 1 month, measurements were no longer significantly different from preoperative values at either 1 mm or 3 mm. From two months onward, thickness values remained comparable to or slightly below baseline, without statistically significant differences. Notably, at 6–12 months, mean conjunctival thickness tended to be lower than preoperative levels (−11.8% to −23.2% at 1 mm), although these reductions did not reach statistical significance. The decrease was more pronounced at 1 mm compared with the thickness at 3 mm (Figure 2, Table 3).

Because multiple time-point comparisons were performed, the results were rechecked using Tukey’s multiple-comparison procedure. At 1 mm from the anterior edge of the EPP, conjunctival thickness at postoperative day 4 and 2 weeks remained significantly greater than baseline (*p* = 0.0235 and 0.0363, respectively), whereas the difference between baseline and 1 month or later were not significant (*p* > 0.5). Similarly, at 3 mm, conjunctival thickness at postoperative day 4 and 2 weeks remained significantly greater than baseline (*p* = 0.0023 and 0.0390, respectively), whereas, no significant difference was found at 1 month or later (*p* > 0.5).

The intraclass correlation coefficients (ICCs) for conjunctival thickness measurements were exceptionally high, measuring 0.999 at 1 mm and 0.997 at 3 mm from the anterior edge of the EPP indicating excellent measurement reproducibility.

Patch extrusion occurred in three eyes in the EPP group at 2.6, 5.2, and 8.9 months postoperatively. In the scleral patch graft group, extrusion occurred in 12 eyes at 0.6, 1.0, 1.6, 1.7, 1.8, 2.5, 3.2, 3.3, 6.5, 7.7, 8.2, 10.9 months. Eyes without extrusion were censored at their last follow-up visit, with a maximum follow-up duration of 12 months.

### 3.2. Propensity Score Overlap Weighting and Covariate Balance

Propensity scores were estimated using age, neovascular glaucoma (NVG; yes/no), history of prior ocular surgery, preoperative intraocular pressure, number of preoperative glaucoma medications, surgical quadrant (supero-temporal vs. others), and insertion site (pars plana insertion vs. others).

The type of glaucoma drainage device was not included in the PS model because only Ahmed glaucoma valves were used in the control group, resulting in a violation of the positivity assumption.

After applying overlap weighting, covariate balance between the EPP and scleral patch graft groups improved substantially. Standardized mean differences (SMD) were substantially reduced across covariates (Table 4). Although small residual imbalance persisted for age, NVG, and insertion site, overall balance in the weighted pseudo-population was considered acceptable. Examination of the propensity score and weight distributions confirmed adequate overlap between groups without evidence of extreme weights.

**Figure 3:**
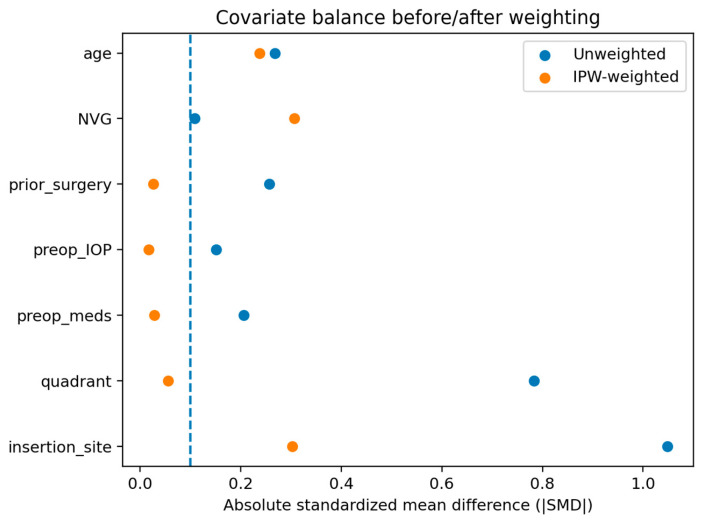
Covariate balance before and after propensity score weighting. Dotted line indicates the threshold for optimal standardized mean differences (SMD) of 0.1.

Love plot illustrating the absolute standardized mean differences (SMDs) for baseline covariates before and after overlap weighting. After weighting, substantial reduction in SMD was observed across covariates, indicating improved balance between the EPP and scleral patch graft groups (Figure 3).

**Figure 4:**
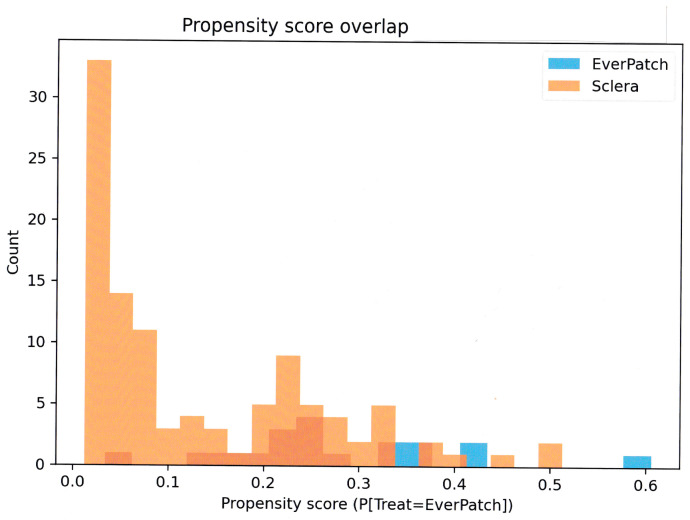
Propensity score distribution and overlap. Overlapping regions of the orange and blue histograms appear as dark orange, indicating area of common support between groups.

Histograms showing the distribution of propensity scores in the EPP and scleral patch graft groups. Adequate overlap between groups was observed, supporting the appropriateness of overlap weighting for comparative time-to-event analysis (Figure 4).

### 3.3. Propensity Score Weighting and Survival Analysis

To adjust for baseline differences between the EPP and scleral patch graft groups, a propensity score (PS) for treatment assignment was estimated using multivariable logistic regression.

Stabilized inverse probability of treatment weights (IPW) for the average treatment effect were calculated as *P*(*Treatment*)/*PS* for the EPP group and (1 − *P*(*Treatment*))/(1 − *PS*) for the scleral patch graft group. Covariate balance before and after weighting was assessed using standardized mean differences (SMDs), with smaller absolute SMD indicating improved balance.

Because the maximum follow-up duration in the EPP group was limited to 12 months, all survival analyses incorporated administrative censoring at 12 months. Extrusion-free survival was evaluated using both unweighted and IPW-weighted Kaplan–Meier methods (the unweighted Kaplan–Meier survival curve is not shown). Between-group differences were assessed using the log-rank test. A two-sided *p* value < 0.05 was considered statistically significant.

After inverse probability weighting and administrative censoring at 12 months, the estimated 12-month extrusion-free survival was 88.4% (95% CI, 82.1–94.8%) in the scleral patch group and 83.5% (95% CI, 67.8–99.2%) in the EPP group. In the weighted analysis, each eye contributed to the Kaplan–Meier estimator according to its propensity score-derived weight.

**Figure 5:**
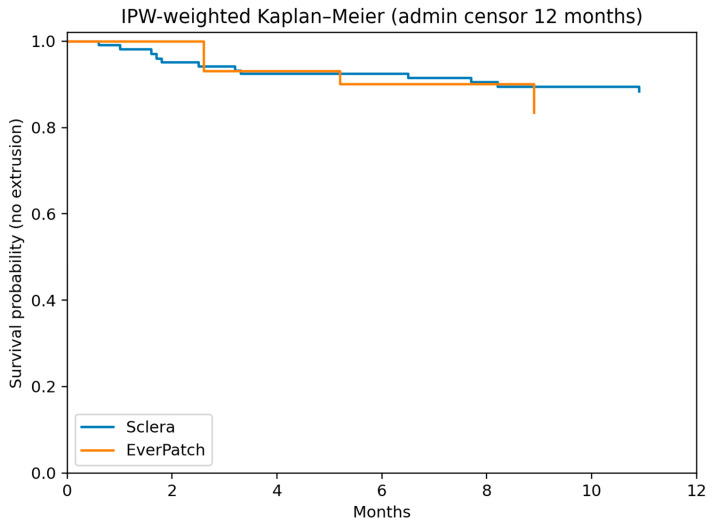
Inverse probability-weighted (IPW) Kaplan–Meier curve demonstrating extrusion-free survival following glaucoma drainage device surgery using EPP (EverPatch group) and conventional scleral patch grafts (scleral group).

There was no significant difference in extrusion-free survival between groups (Figure 5: log-rank test, *p* = 0.498).

### 3.4. IPW-Weighted Cox Proportional Hazards Model

As a sensitivity analysis, an inverse probability-weighted Cox proportional hazards model was fitted to estimate the hazard ratio (HR) for patch graft extrusion associated with EPP use.

In an IPW-weighted Cox proportional hazards model with administrative censoring at 12 months, use of EPP was not significantly associated with an increased risk of patch extrusion compared with scleral patch grafts (hazard ratio, approximately 1.3; 95% confidence interval, approximately 0.4–4.0; *p* = 0.65). Owing to the limited number of extrusion events in the EPP group, the confidence interval around the HR was wide.

Propensity score weighting was applied to adjust for baseline covariates, and administrative censoring was applied at 12 months. No statistically significant difference in extrusion-free survival was observed between groups (log-rank test, *p* = 0.498).

Extrusion of EPP occurred in three eyes. Among the remaining 16 eyes that were followed for 10.7 ± 1.0 months, no abnormal vascular dilatation, conjunctival bleaching, or patch extrusion was observed during follow-up.

To evaluate potential migration of the EPP, the distance between the scleral spur (SS) and the anterior edge of the EPP was measured (Table 5). The measurement obtained at the first posoperative visit (between postoprative day 4 and week 1) was used as the baseline. Eyes in which measurement was not feasible due to peripheral anerior synechiae or poor-quality angle images were excluded from this analysis. The SS-EP distance remained largely stable throughout the follow-up period, and the small increase observed over time were not statistically significant (Table 5).

Conjunctival microcysts, a common finding after filtering surgery, were observed in 8 of 19 eyes. These microcysts resolved spontaneously within one month without sequalae. Eyes with thicker preoperative conjunctivas tended to exhibit more pronounced postoperative edema.

Accumulation of fluid around the patch was observed in 8 of 18 eyes within the first two postoperative weeks. In 7 of these cases, the fluid resolved spontaneously within one month. In the remaining case, fluid accumulation persisted and ultimately progressed to EPP extrusion by the fifth postoperative month. In 6 of 19 cases, neither fluid accumulation nor conjunctival microcyst were observed around the patch material.

At 9 months, conjunctival wound healing was satisfactory in 16 of the 19 eyes (84%), with the EPP completely covered by intact conjunctiva. Conjunctival thickness at 6–12 months was lower than the preoperative thickness; however, this reduction did not reach statistical significance.

**Figure 6:**
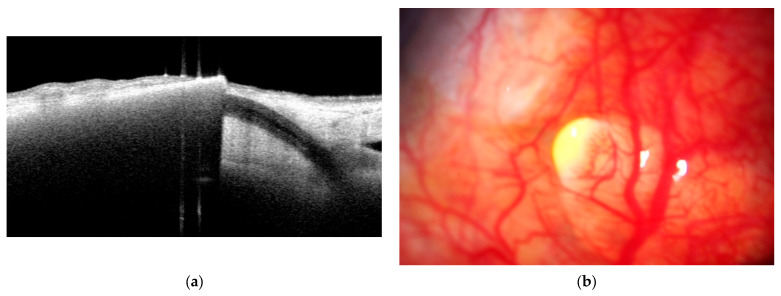
(**a**,**b**) Destruction of the conjunctiva at the margin of EPP. Vertical misalignment or edge lifting of the EPP resulted in disruption of the overlying conjunctiva.

Two distinct patterns of EverPatch extrusion were identified. In one of the three extruded cases, the edge of the EPP mechanically disrupted the overlying conjunctiva leading to exposure of the EPP (Figure 6a,b). The clinical course in this case was comparable to that observed in conventional scleral patch graft-related extrusion.

**Figure 7:**
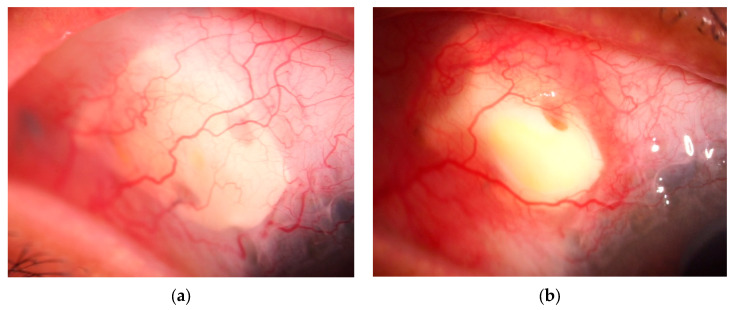
(**a**,**b**) Slit-lamp findings in a case of central-type extrusion. Representative slit-lamp photographs illustrating the clinical course of an EPP extrusion case. (**a**) Appearance at 6.7 months postoperatively. Prior to extrusion, demonstrating decreased vascularity over the anterior central portion of the patch. (**b**) Appearance at 8.9 months postoperatively, showing progression to conjunctival melting and subsequent patch exposure. This type of extrusion began as a small dimple in the central area of EPP and progressed rapidly, leading to conjunctival melting and subsequent exposure of the patch.

**Figure 8:**
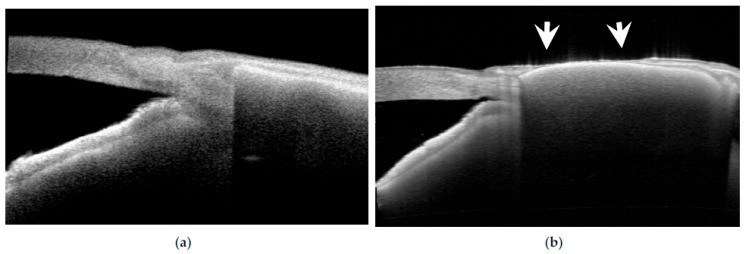
(**a**,**b**) Anterior segment OCT findings in a case of conjunctival melting and subsequent EPP extrusion. Anterior segment OCT images obtained before extrusion at 6.7 months postoperatively (**a**) and after extrusion at 8.9 months postoperatively (**b**). Melting occurred in the central portion of the EPP, corresponding to the area of subsequent extrusion (arrows).

A different pattern of extrusion was observed in 2 of the 19 eyes treated with EPP, characterized by conjunctival bleaching followed by progressive melting of the overlying conjunctiva. The initial ischemic change developed in the anterior central portion of the EPP (Figure 7a,b), followed by progressive melting of the overlying conjunctiva (Figure 8). This pattern of extrusion was not observed in any of the 12 scleral patch graft cases with extrusion, representing a statistically significant difference between groups (*p* = 0.024 Fischer exact test).

During surgical repair with conjunctival rotation, weak adhesion between the conjunctiva and the EPP was observed, and the patch could be easily separated from both the conjunctiva and the underlying sclera, suggesting limited fibrotic integration.

**Figure 9:**
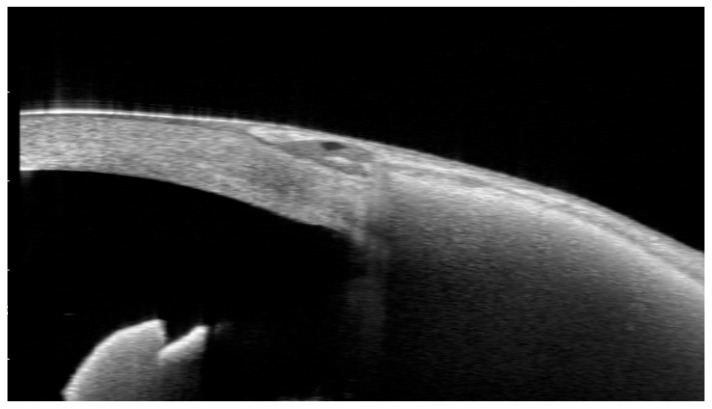
Cyst formation anterior to the EPP at 5.6 months postoperatively. A cystic structure was observed in front of the EPP in 1 of 19 eyes.

In all cases, no abnormal exudate, subconjunctival abscess, or clinical signs of infection were detected, and no patient reported pain or discomfort. These findings suggest the absence of significant inflammatory or infectious reactions associated with the EPP. However, in one case, a cyst-like structure developed anterior to the EPP at 5.6 months postoperatively (Figure 9), indicating localized fluid accumulation without overt inflammatory features.

## 4. Discussion

Recently, the scleral tunnel technique has gained popularity as an alternative approach [6,7]. However, concerns remain regarding the risk of tube exposure with this method, particularly in patients younger than 65 years of age [19]. Therefore, patch grafting may continue to represent the mainstream method for tube coverage.

Previously reported risk factors for tube or GDD exposure include the number of prior surgeries [20,21,22,23], number of medications [24], patient age [20,22,25], quadrant of implantation [24,26], preoperative inflammation [25], use of non-valved implants [23,27], diabetes [25,27], combined surgery [22,28], White race [29], female sex [20,29], and the use of pericardial patch grafts [30].

Previously reported rate of GDD exposure in adults range from 3% to 8% during the first 1–5 years after surgery [29]. However, the proportion of extrusion is likely to increase when a higher percentage of eyes have undergone multiple prior surgeries or when devices are implanted in the inferior quadrant; therefore, substantial inter-institutional variability can be expected. In the primary tube versus trabeculectomy (PTVT) study, which evaluated primary surgeries, the one-year extrusion rate was only 1% [31]. In the present study, the incidence of extrusion was relatively high in both the control and EPP groups. This may be attributable to the high proportion of reoperated cases and the frequent placement of implants in the inferior quadrant. In addition, specific patient characteristics at our institution—as well as regulatory restrictions in Japan that preclude the use of processed preserved sclera such as Tutoplast, thereby necessitating the use of fresh sclera—may have contributed to the higher extrusion rate [27]. Given this background, minimizing the influence of confounding factors is essential; therefore, adjustment using weighted propensity scores, as performed in this study, is important. Under these conditions, the extrusion rate of EPP was not significantly different from that of the control group (*p* = 0.498, log-rank test).

Although an extrusion rate of 3/19 eyes (15.8%) at 10.7 ± 1.0 months is by no means low, it remains markedly lower than the 48.3% rate (13/27 eyes within 5 months) previously reported [18]. This marked discrepancy may, at least in part, be attributable to differences in surgical technique.

The pore size of woven PCU has been reported to be approximately 3.7 µm, which is likely too small to permit fibroblast infiltration [18]. In addition, the hydrophobic nature of polycarbonate urethane may result in poor adhesion between the conjunctiva and the EPP facilitating migration over the scleral surface. For this reason, the EPP should be firmly secured to the sclera using nonabsorbable sutures. The use of only two absorbable sutures, which is not recommended in conventional GDD surgery [32,33], may contribute to a higher incidence of patch migration and subsequent extrusion in EPP cases [18]. In the present study, the EPP was firmly anchored with four nylon sutures, and migration was minimal (Wilcoxon signed rank test, *p* > 0.1, Table 5). These technical differences, particularly the use of multiple nonabsorbable fixation sutures and meticulous conjunctival closure, may explain the substantially lower extrusion rate observed in our series compared with the higher rate reported previously.

The EPP is very thin, measuring only 100 to 150 μm, approximately four to five times thinner than the sclera and is therefore less likely to protrude. However, vertical misalignment or edge lifting may occur if it is inadequately sutured to the sclera. Mechanical stress caused by lifting of the EPP against the overlying conjunctiva may contribute to conjunctival breakdown at the patch margin. If a large patch is insufficiently fixed, distortion or focal protrusion may generate focalized stress on the overlying conjunctiva. Improvement in patch design, downsizing, and refinement of surgical application techniques may further enhance tissue compatibility and reduce mechanical complications.

In contrast to mechanical conjunctival damage, a different mechanism may underly extrusion in certain EPP cases. In this series, two of the three eyes with extrusion exhibited conjunctival melting followed by extrusion, a pattern not observed in the 12 extruded cases using conventional biological patch grafts (Figure 7 and Figure 8). In these cases, there was no apparent elevation or prominence at the site of breakdown. Rather, the process began as a small dimple near the center of the patch approximately six months postoperatively and then progressed rapidly.

Large, oxygen-impermeable patches may impede oxygen and nutrition diffusion to the overlying conjunctiva. In such circumstances, the conjunctiva depends primarily on intra-conjunctival vessels that stem from surrounding tissue.

As the conjunctiva overlying the patch becomes progressively thinner and more attenuated over time, vascular supply may decrease. Eyes with preoperatively thin conjunctiva or conjunctiva compromised by prior surgeries may therefore be at greater risk of erosion. In biological patch graft, vascular ingrowth into the graft material may help support oxygen and nutrient delivery to the overlying conjunctiva; however, such integration is unlikely with EPP patching. Bleaching of the overlying conjunctiva may represent an early sign of ischemia and precursor to tissue erosion. Thus, in addition to mechanical compression and tissue atrophy, local ischemia and impaired nutritional support may contribute to extrusion. Accordingly, the use of a smaller patch may help mitigate this risk.

### Tissue Reaction

The EPP is composed of hydrophobic PCU, which differs fundamentally from traditional biological patch grafts such as sclera or pericardium, both of which are hydrophilic. The absence of vasodilation, overt inflammation, or patient discomfort argues against cytotoxicity or a clinically significant foreign body reaction. The fluid accumulation and conjunctival microcysts observed between postoperative day 4 and 2 weeks resolved spontaneously within 1 month. Similar transient fluid accumulations have been reported in eyes receiving conventional hydrophilic pericardial patch grafts [4].

Because GDD implantation functions as a form of filtering surgery, it is plausible that aqueous humor pooling over the implant plate or leakage from the tube’s insertion site may result in temporary accumulation of aqueous humor around the EPP. Therefore, these findings are unlikely to represent EPP-specific events. Biological materials may deposit onto the hydrophobic surface of the EPP, potentially converting it into a more hydrophilic surface over time [18]. If this process is excessive, cystic changes may develop (Figure 9). However, the absence of vasodilation suggests that any associated tissue response is minimal, if present at all.

Taken together with the progressive thinning of the postoperative conjunctiva (Figure 2), these observations indicate a minimal inflammatory response, low cytotoxicity, and limited foreign body reaction associated with polycarbonate urethane. Whether progressive conjunctival thinning predisposes to late melting or future erosion remains to be determined and warrants future investigation.

## 5. Limitations

This study has several limitations. First, the follow-up duration in the EPP group was limited to 12 months. Because patch extrusion is a time-dependent complication that may occur beyond the first postoperative year, longer follow-up is necessary to fully evaluate the long-term safety profile of EPP. To mitigate the imbalance in follow-up duration between groups, we performed survival analyses with administrative censoring at 12 months and applied propensity score-based inverse probability weighting. Nevertheless, the possibility of late-onset extrusion events cannot be excluded. Second, the sample size of the EPP cohort was relatively small, which may limit statistical power and precision of effect estimates. Third, our PS model was restricted to variables consistently available in all eyes (age, prior surgery, preoperative IOP, glaucoma subtype, and medication burden). Therefore, residual confounding from unmeasured factors (e.g., type of device, conjunctival status, and surgeon-specific techniques) cannot be excluded. Finally, although inverse probability weighting substantially improved covariate balance, some residual imbalance remained for age, neovascular glaucoma, and insertion site, which should be considered when interpreting the results. A study with longer follow up and larger sample sizes is warranted.

## 6. Conclusions

The EverPatch Plus^®^ (EPP), a synthetic patch graft fundamentally different from traditional biological materials, was used to cover tubes in 19 glaucoma drainage device surgeries. Postoperatively, conjunctival thickness increased by 81.9–110.8% at 4 days and returned to preoperative levels within 1–2 months. No exudation or clinically significant inflammation was observed around the artificial patch graft. After inverse probability weighting and administrative censoring at 12 months, the estimated 12-month extrusion-free survival was 88.4% in the scleral patch group and 83.5% in the EPP group, with no significant difference between groups (log-rank test, *p* = 0.498). These findings suggest that the short-term safety profile of EPP is comparable to that of conventional scleral patch grafts. However, conjunctival melting preceding extrusion may represent a distinct complication associated with EPP, warranting careful long-term observation and further investigation.

## Figures and Tables

**Table 1:** Demographic data of patients treated with EverPatch and scleral patch.

	EverPatch	Scleral Patch	*p* Value (ANOVA)
Age	67.1 ± 15.6	62.5 ± 17.9	0.305
Type of Glaucoma	NVG 5, POAG 8, PEG 2, SG 2, PACG 2	NVG 27, POAG 25, PEG 10, SG 26, PACG 5	0.092 (residual analysis)
Sex: Male/Female	12/7	57/48	0.617
Side: R/L	9/10	50/55	1.00
Pre-op IOP (mmHg)	31.5 ± 12.8	29.4 ± 9.4	0.521
Pre-op BCVA (logMAR)	0.980 ± 0.960	0.708 ± 0.857	0.213
Pre-op meds	4.4 ± 1.2	4.1 ± 1.2	0.409
Number of prior surgeries	2.4 ± 1.7	2.7 ± 1.9	0.444
Visual field defects (HFA: MD, dB)	−20.6 ± 10.9 *	−18.9 ± 9.3 *	0.556
Type of GDD used	AGV 6, PGI 13	AGV 105	NA
Type of EverPatch	Shield type 16, Rectangular type 3	NA	
Quadrant of tube insertion	Sup-temporal 16, Inf-nasal 3	Sup-temporal 52, Sup-nasal 12, Inf-temporal 30, Inf-nasal 11	0.005 (residual analysis)
Tube tip location	Anterior chamber 6, Ciliary sulcus 11, pars plana 2	Anterior chamber 26, ciliary sulcus 21, pars plana 58	*p* < 0.001 (residual analysis)
Pre-op conjunctival thickness at 1 mm from SS	0.262 ± 0.100	NA	
Pre-op conjunctival thickness at 3 mm from SS	0.267 ± 0.114	NA	

NVG: Neovascular glaucoma, POAG; primary open angle glaucoma, PEG; exfoliation glaucoma, SG; secondary glaucoma, PACG; primary angle closure glaucoma, *: visual field data of 4 patients in EverPatch cohort and 48 patients in control was not available due to severe visual disturbance. Pre-op: preoperative, BCVA (logMAR): best corrected visual acuity by logarithmic minimal angular resolution. HFA MD: mean deviation assessed by Humphrey visual field analyzer, dB: decibels. GDD: glaucoma drainage device, AGV: Ahmed Glaucoma valve, PGI: Paul Glaucoma Implant, SS: scleral spur.

**Table 2:** Summary of postoperative IOP and medications.

		Pre-Op	1 M	3 M	6 M	12 M
EverPatch	# of medications	4.4 ± 1.2	NA	NA	NA	2.6 ± 1.8
	IOP	31.5 ± 12.8	16.7 ± 4.3	15.2 ± 6.0	14.8 ± 4.3	15.4 ± 4.8
Control	# of medications *p* by ANOVA	4.1 ± 1.2 *p* = 0.409	NA	NA	NA	2.2 ± 1.5 *p* = 0.379
	IOP	29.7 ± 10.9	19.7 ± 9.0	18.1 ± 5.7	16.9 ± 4.8	16.4 ± 5.9
	*p* by ANOVA	0.521	0.17	0.041	0.075	0.549

IOP: intraocular pressure, logMAR: logarithms minimal angle resolution, BCVA: best corrected visual acuity.

**Table 3:** Trends in conjunctival thickness following patching with EverPatch Plus.

Time Point	1 mm Thickness (mm)	% Change vs. Baseline	*p* vs. Baseline	3 mm Thickness (mm)	% Change vs. Baseline	*p* vs. Baseline
Baseline	0.262 ± 0.100	0	-	0.267 ± 0.114	0	-
4 days *n* = 19	0.477 ± 0.239	81.9	0.002 ↑	0.562 ± 0.333	110.8	0.001 ↑
2 weeks *n* = 18	0.471 ± 0.221	79.5	0.001 ↑	0.509 ± 0.262	90.9	0.001 ↑
1 month *n* = 19	0.311± 0.16	18.8	0.756 (ns)	0.357 ± 0.170	33.7	0.077 (ns)
2 months *n* = 19	0.250 ± 0.149	−4.6	0.779 (ns)	0.297 ± 0.192	11.3	0.578 (ns)
3 months *n* = 18	0.245 ± 0.181	−6.0	0.731 (ns)	0.280 ± 0.172	4.9	0.793 (ns)
6 months *n* = 19	0.218 ± 0.154	−16.7	0.731 (ns)	0.245 ± 0.159	−8.2	0.660 (ns)
9 months *n* = 17	0.231 ± 0.237	−11.8	0.593 (ns)	0.224 ± 0.232	−16.0	0.465 (ns)
12 months *n* = 9	0.201 ± 0.091	−23.2	0.278 (ns)	0.249 ± 0.140	−6.5	0.797 (ns)

↑ = significantly greater than comparator; ns = not significant.

**Table 4:** Standardized mean differences (SMD) for all covariates.

Covariate	SMD Unweighted	SMD Weighted
Age	0.268543	−0.23788
NVG	−0.10863	0.306316
prior surgery	−0.25719	0.026397
Preop IOP	0.15107	−0.01721
Preop meds	0.205732	−0.02854
Quadrant	0.782763	0.055892
Insertion site	−1.04855	−0.30277

SMD: Standardized mean differences.

**Table 5:** Trends in the distance between the scleral spur (SS) and the anterior edge of the EPP.

Time Point	SS-EPP (mm)	*p* *
4 days *n* = 9	1.01 ± 0.20	
2 weeks *n* = 7	1.07 ± 0.23	0.893
1 month *n* = 14	1.13 ± 0.34	0.263
2 months *n* = 15	1.20 ± 0.39	0.953
3 months *n* = 14	1.21 ± 0.40	0.484
6 months *n* = 14	1.17 ± 0.532	0.499
9 months *n* = 17	1.23 ± 0.542	0.214
12 months *n* = 8	1.19 ± 0.643	0.144

SS: scleral spur, *p* *: *p* value by Wilcoxon signed-rank test comparing day 4 or one-week data with each corresponding time point.

## Data Availability

The research data used in this research are available upon request from the corresponding author.

## Abbreviations

The following abbreviations are used in this manuscript:

EPP	EverPatch Plus^®^
GDD	Glaucoma drainage device
PCU	Polycarbonate urethane
FDA	Food and Drug Administration
AS-OCT	Anterior segment optical coherence tomograph
PS	Propensity score
SMD	Standardized mean difference
IPW	Inverse probability of treatment weighting
HR	Hazard ratio
CI	Confidence interval
AGV	Ahmed glaucoma valve
PGI	Paul glaucoma implant
ANOVA	Analysis of variance
IOP	Intraocular pressure
NVG	Neovascular glaucoma
POAG	Primary open angle glaucoma
PACG	Primary angle closure glaucoma
SG	Secondary glaucoma
Log MAR	Logarithmic minimal angular resolution
BCVA	Best corrected visual acuity
HFA	Humphrey visual field analyzer
MD	Mean deviation
dB	Decibel
SS	Scleral spur
